# IL-8^+^ neutrophils drive inexorable inflammation in severe alcohol-associated hepatitis

**DOI:** 10.1172/JCI178616

**Published:** 2024-03-19

**Authors:** Yukun Guan, Brandon Peiffer, Dechun Feng, Maria A. Parra, Yang Wang, Yaojie Fu, Vijay H. Shah, Andrew M. Cameron, Zhaoli Sun, Bin Gao

**Affiliations:** 1 Laboratory of Liver Diseases, National Institute on Alcohol Abuse and Alcoholism, NIH, Bethesda, Maryland, USA.; 2 Department of Surgery, Johns Hopkins University School of Medicine, Baltimore, Maryland, USA.; 3 Department of Medicine, Mayo Clinic, Rochester, Minnesota, USA.

**Keywords:** Hepatology, Hepatitis, Innate immunity, Neutrophils

**To the Editor:** Alcohol-associated liver disease (ALD), one of the major chronic liver diseases worldwide, includes a spectrum of liver disorders from simple steatosis to steatohepatitis and cirrhosis. Patients with chronic ALD may develop alcohol-associated hepatitis (AH) with major clinical signs such as jaundice (1). Especially in those with alcohol-associated cirrhosis (AC), severe AH (sAH) has high short-term mortality and lack effective pharmacological therapies (1). To explore the mechanisms underlying the transition from AC to sAH, we performed single cell RNA-Seq (scRNA-Seq) analysis of livers and peripheral WBC from sAH and AC patients. We also included the published scRNA data of the livers of 5 healthy donors and of 2 patients with AC from the GEO database (2) in our analysis. We identified 15 clusters of cell populations using 30 principal components under the resolution of 0.1, with most of the cells being immune cells (Figure 1A and Supplemental Figure 1, A–D; supplemental material available online with this article; https://doi.org/10.1172/JCI178616DS1**)**. Intriguingly, we found that the major difference between sAH and AC was that sAH livers had a markedly higher number of neutrophils (Cluster 0) than AC livers, while the differences in other subsets of immune cells between AC and sAH were less evident (Figure 1A), suggesting that hepatic neutrophil infiltration is an important factor promoting AC to sAH transition.

Next, we further focused on the neutrophil cluster. We found two distinct liver sAH-specific neutrophil clusters, 2 and 4, and a circulating sAH-specific neutrophil cluster, 3 (Figure 1B and Supplemental Figure 1C). The sAH-specific liver neutrophil clusters 2 and 4 exhibit unique gene profiles notably characterized by heightened expression of *CXCL8*, a gene encoding IL-8, defined as IL-8*^+^* neutrophils (Figure 1C and Supplemental Figure 1C). The gene expression pattern including transcription factor–related genes in clusters 2 and 4 is different from that in other clusters (Supplemental Figure 2A). Pathway analysis revealed that top three differentially expressed hallmark pathways in liver IL-8^+^ neutrophils are related to TNF-α, IFN-γ, and inflammatory response, compared with those in circulating neutrophils, where there is upregulation of TNF-α and inflammatory genes but downregulation of IFN-related genes (Supplemental Figure 2, B and C). We then conducted immunostaining to validate sAH-specific liver IL-8^+^ neutrophils identified by scRNA-Seq. Figure 1D shows that sAH livers exhibited a much greater number of neutrophils, with most of these neutrophils displaying higher levels of IL-8 expression than those in AC livers. Further staining of IL-8 in isolated liver nonparenchymal cells (NPCs) and WBCs showed that sAH WBCs had approximately 20% IL-8^+^ cells, while IL-8^+^ cells were barely detected in AC WBCs, and sAH liver NPCs had approximately 70% IL-8^+^ cells, while only approximately 5% IL-8^+^ cells were detected in AC NPCs (Supplemental Figure 3A). As IL-8 is a key chemokine for neutrophil activation (3), accumulation of IL-8^+^ neutrophils in sAH likely contributes to self-sustained neutrophil activation and liver inflammation in these patients.

To understand the mechanisms underlying upregulation of IL-8 in infiltrating neutrophils in sAH, we analyzed the genes (such as IL-1β and TNF-α) and transcription factors that are known to upregulate IL-8 (3), in neutrophils and liver tissues. Our scRNA-Seq data revealed that neutrophil populations expressed higher levels of *IL-1R* and *TNFR* genes than other cell populations (Supplemental Figure 3B), specifically in the IL-8^+^ neutrophils, clusters 2 and 4, which highly express TNFRSF1A/B and IL1R1/2, respectively (Figure 1E). The transcription factors, REL and FOS, that are known to upregulate IL-8 gene expression (3) were detected at higher levels in IL-8^+^ clusters 2 and 4 than in other clusters (Figure 1E). Moreover, our bulk RNA-Seq data in Supplemental Figure 3C, when comparing sAH with healthy donor and AC livers, revealed marked upregulation of genes related to the IL-1 and TNF families and their associated receptors. Finally, activated/phosphorylated p38MAPK, an important signaling pathway that activates IL-8 (3), was detected in sAH liver neutrophils (Figure 1F and Supplemental Figure 3D).

To understand the mechanism underlying neutrophil recruitment to sAH but not AC livers, we measured the mRNA and protein levels of neutrophil chemokines in the liver. We found that most of these chemokines were higher in sAH livers compared with levels in healthy and AC livers, with the highest fold elevation of IL-8 in sAH patients (Supplemental Figure 4, A and B). We then explored the cell types that express these chemokines by analyzing our scRNA-Seq data. We observed *CXCL8* in neutrophils, *CXCL6* in hepatocytes, and *CXCL5* in hepatic stellate cells, while *CXCL1* was detected in both hepatocytes and neutrophils (Figure 1G). Such expression patterns were further confirmed by multiplex staining (Supplemental Figure 4C).

In conclusion, although the elevation of IL-8 and neutrophils in AH has been known for many years (1), the targeting of neutrophils has not been tested in the clinic for patients with sAH, and the roles of IL-8 and neutrophil elevation in AH pathogenesis remain underexplored and warrant further investigation. Our current study has demonstrated that patients with sAH had self-sustaining IL-8^+^ neutrophil accumulation, which likely drives inexorable liver inflammation and failure. Targeting IL-8^+^ neutrophils could be a promising therapeutic strategy for sAH by directly blocking IL-8 signaling — via anti-IL-8 antibodies or CXCR1/2 antagonists — or indirectly blocking the inflammatory signals inducing IL-8 (3).

## Supplementary Material

Supplemental data

Supporting data values

## Figures and Tables

**Figure 1:**
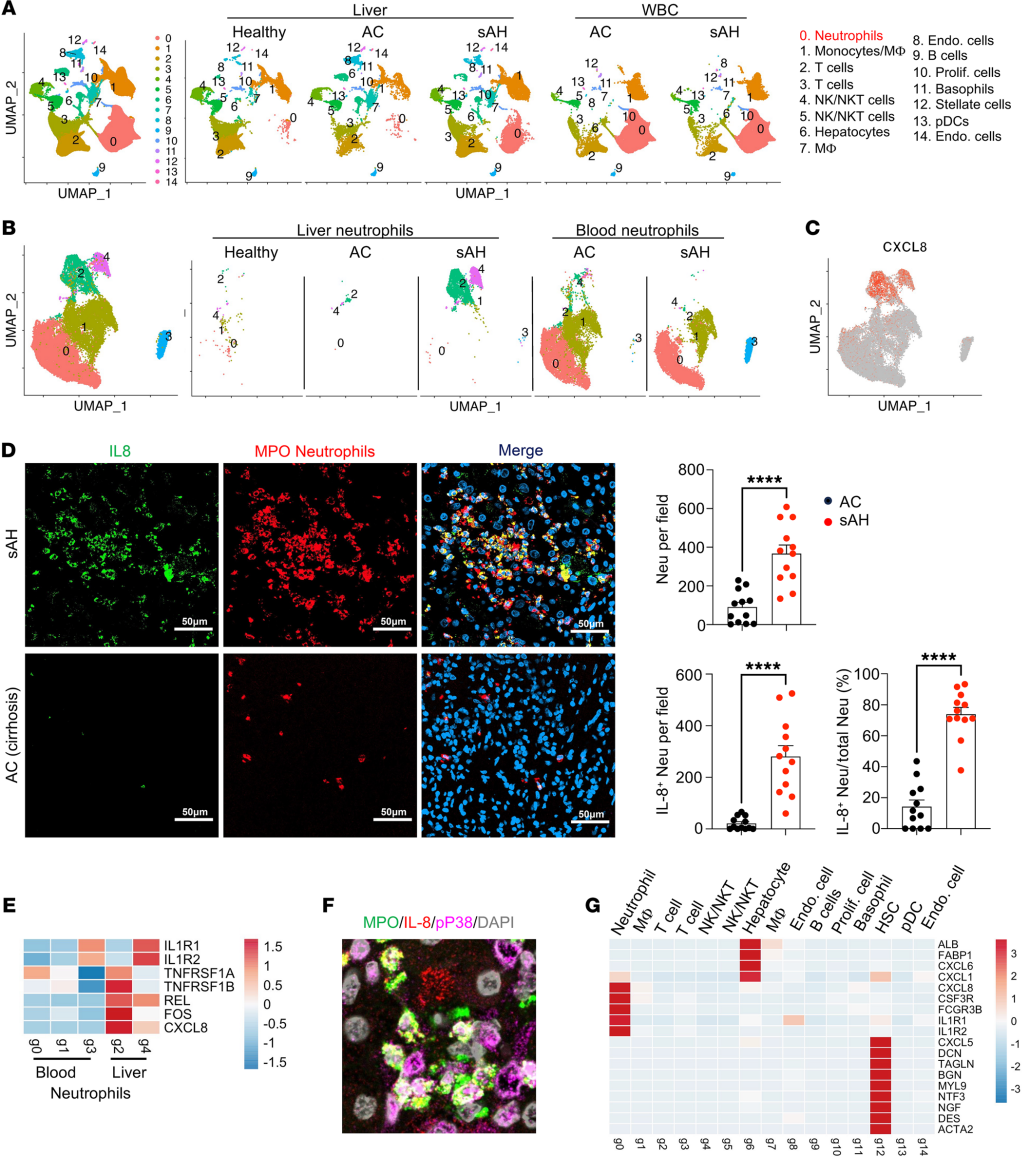
sAH is associated with an accumulation of self-sustaining IL-8^+^ neutrophils. (**A**) scRNA sequencing data of liver cells from 5 healthy donors — 3 AC and 5 sAH — and peripheral WBC from 3 donors with AC and 4 donors with sAH. In total, 87,105 cells were analyzed, integrated, and clustered by Seurat. UMAP plots of each group are shown. (**B**) Neutrophils (CD16^+^CD114^+^, Cluster 0 in Figure 1A) from all groups were reclustered. UMAP plots of all the neutrophils from each group are shown. (**C**) Feature plots for the gene expression of *CXCL8* (a gene encoding IL-8 protein) among all groups of neutrophils. (**D**) sAH and AC liver tissues were stained with IL-8 and neutrophil marker MPO. Representative images are shown. The number and percentage of IL-8^+^ neutrophils are presented as means ± SEM. ****P < 0.0001. Scale bars: 50 μm. (**E**) Heatmap of genes that related to IL-8 upregulation in neutrophils from scRNA-Seq data. (**F**) sAH liver tissues were stained with IL-8, phospho-p38 MAPK, and neutrophil marker MPO. A representative merged image is shown, and the image with staining with each individual antibody is shown in Supplemental Figure 3D. Scale bars: 20 μm. (**G**) Heatmap of neutrophil chemokine and cell maker genes from scRNA-Seq data.

## Version 1 03/19/2024

In-Press Preview

## Version 2 05/01/2024

Electronic publication
