# Supplementary material for: IL-8+ neutrophils drive inexorable inflammation in severe alcohol-associated hepatitis
Source: J Clin Invest. 2024 Mar 19;134(9):e178616. doi: 10.1172/JCI178616 (PMC11060723; doi:10.1172/JCI178616)
Supplement: Supplemental data [file jci-134-178616-s231.pdf]

Supplemental Materials

IL-8⁺ neutrophils drive inexorable inflammation in severe alcohol-associated hepatitis

Yukun Guan[1*], Brandon Peiffer[2*], Dechun Feng[1*], Maria A. Parra[2], Yang Wang[1], Yaojie Fu[1], Vijay H. Shah[3], Andrew M. Cameron[2], Zhaoli Sun[2], Bin Gao[1]

[1]Laboratory of Liver Diseases, National Institute on Alcohol Abuse and Alcoholism, NIH, Bethesda, Maryland, USA. [2]Department of Surgery, Johns Hopkins University School of Medicine, Baltimore, Maryland, USA. [3]Department of Medicine, Mayo Clinic, Rochester, Minnesota, USA.

*****YG, BP, and DF contributed equally to this work.

Address correspondence to: Bin Gao, NIAAA, NIH, 5625 Fishers Lane, Bethesda, Maryland 20892, USA. Email: bgao@mail.nih.gov. Or to: Zhaoli Sun, Johns Hopkins University School of Medicine, 720 Rutland Avenue, Baltimore, Maryland 21205, USA. Email: zsun2@jh.edu.

Research Support

This work was supported by the intramural program of the NIAAA (BG) and the NIH R24AA025017 and P50AA027054 (ZS).

Author Contributions

Y.G., B.P., D.F. designed and performed experiments, analyzed the data, and edited the manuscript. Y.G. wrote the manuscript. M.P, Y.W, Y.F. performed some experiments and data analysis, and edited the paper. V.H.S. and A.M.C helped with data analysis, provided relevant intellectual input, and edited the manuscript. Z.S. and B.G. obtained funding, supervised the whole project, and wrote the paper. All authors approved the final manuscript.

Conflict of Interest

All authors declare no competing interests.

Supplemental Result Section

Hepatocyte subset (cluster 6 in the Fig 1A) was further analyzed. A limited number of cells that express hepatic, biliary, and progenitor cell markers were re-clustered and analyzed (Supplemental Figure S1D). Compared to sAH samples, AC liver samples have a higher number of cells in population 0 that express biliary and progenitor cell marker (1). Interestingly, sAH liver samples have an increased number of unique populations 2 and 3 that express low levels of hepatic, biliary, progenitor cell markers. Further studies are required to identify whether these unique populations in sAH are related to the stimulation from IL-8^{+} neutrophils.

Supplemental Discussion

Targeting IL-8^{+} neutrophils for the treatment of sAH.

Given their important roles in driving inexorable inflammation in sAH, IL-8^{+} neutrophils may be effective therapeutic targets for the treatment of sAH. There are several potential strategies that can be applied to block IL-8^{+} neutrophils.

First, anti-IL-8 antibodies can be used to directly inhibit IL-8. Anti-IL-8 antibodies (ABX-IL-8, HuMax-IL-8/BMS-986253) are currently used in several Phase I/II clinical trials for the treatment of cancers and inflammatory diseases (2), but have not been tested in sAH patients.

Second, CXCR1 and CXCR2 are two important chemokine receptors for IL-8 (2), inhibition of CXCR1/CXCR2 using short lipopeptides (pepducins) has been shown to ameliorate mild alcohol-associated steatohepatitis in mouse models (3). Thus, targeting CXCR1/2 may be an effective strategy for the treatment of sAH. Many CXCR1/CXCR2 antagonists are currently under the investigation of various stages of clinical trials for the

treatment of cancers, diabetes, inflammatory diseases (2). Some of these antagonists could be tested clinically for sAH therapy.

Third, inhibition of IL-8 expression may be another strategy for sAH treatment. IL-8 expression can be blocked by inhibiting the inflammatory environment and/or signals (such as p38 MAPK) that induce IL-8 expression. However, more preclinical studies are required to identify these key inflammatory mediators and signals that drive IL-8 expression in sAH, and test whether blockage of them will have benefits for sAH.

It is well known that bacterial infection is often associated with sAH. Thus, bacterial infection should be closely monitored when the anti-IL-8 or anti-CXCR1/2 therapy is used for sAH. Combination therapy of IL-8 inhibition with antibiotics or IL-22 (a cytokine has strong anti-bacterial functions) (4) may help reduce bacterial infection.

Supplemental Material and Methods

Human samples.

We performed scRNA sequencing analysis of 6 liver tissues and 7 peripheral white blood cells (WBC) from sAH and AC patients, including 5 sAH livers, 4 sAH WBCs; 1 AC liver, and 3 AC WBCs. We measured chemokines in 6 sAH liver samples, 5 AC liver samples and 5 healthy liver samples. We performed multiplex immunofluorescence staining of paraffin-embedded sAH liver tissues which was described previously (5).

The diagnosis of AH and AC is based on previously published guidelines (6, 7). All patients with sAH had cirrhosis or advanced fibrosis. All human samples were received from the Department of Surgery at Johns Hopkins Hospital (supported by the NIAAA, R24AA025017, Clinical resources for AH investigators). The use of liver and serum samples from patients with sAH, AC, and donor controls was approved by the Institutional Review Board (IRB00107893 and IRB00154881) at Johns Hopkins University.

Sex as a biological variable.

Both male and female patients were included in the study.

Multiplex Immunofluorescence staining.

Liver tissues were obtained and fixed in 4% PFA and then embedded in paraffin. Paraffin-embedded sections or Cytospin slides were heated in epitope retrieval citrate buffer (pH 6.0); stained with primary antibodies and fluorescent labeled secondary antibodies (CST); and visualized by LSM 900 Confocal Microscope (Zeiss, Thornwood, NY). Multiplex immunofluorescence staining with more than three markers was performed as previously described (5). Acquired images were processed and analyzed using Image J FIJI (8).

Antibodies.

Antibody	Vendor	Cat. No	Dilution
IL-8	CST	94407	1:200
CXCL1	Thermo Fisher Scientific	PA5-86508	1:200
CXCL5	Thermo Fisher Scientific	710010	1:200
CXCL6	Thermo Fisher Scientific	PA5-67571	1:200
MPO	Developmental Studies Hybridoma Bank	CPTC-MPO-1	1:100
α-SMA	CST	56856	1:200
pP38	CST	4511	1:200
HepPar1	Novus Biologicals	NBP2-45272	1:200

Single-cell RNA sequencing.

Liver cells (6 samples) and WBCs (7 samples) were isolated from consented sAH and AC patients, including 5 sAH livers, 4 sAH WBCs; 1 AC liver, 3 AC WBCs. Venous blood was procured into EDTA-coated tubes. Within one hour of collection, red blood cells were lysed using ammonium chloride buffer (1:20, BioLegend®) for 10 minutes and the resulting WBCs were washed twice with PBS. Thin (1cm) liver sections were taken from the right lobe of explanted livers. The tissues were coarsely minced before 1 wash with PBS to remove excess red blood cells and albumin. The washed tissues were suspended in a 0.1% collagenase/0.025% hyaluronidase solution and incubated at 37°C with constant agitation for 30 minutes. After incubation, tissue homogenates were centrifuged at 50g for 2 minutes to pellet debris and the resulting supernatant was collected and washed with PBS. Using OptiPrep® and DMEM, the density of the cell pellet was adjusted to ρ=1.5 and floated through discontinuous layers of ρ=1.2 and PBS. Viable liver cells were collected from the ρ=1.2/PBS interface, washed with PBS.

Liver cells (6 samples) and WBCs (7 samples) from AC and sAH patients were loaded in the lanes using 10X Genomics Chromium Next GEM Single Cell 3' Kit v3.1 with a single capture lane per sample targeting recovery of 8,000 cells. All subsequent steps of library preparation and quality control were performed according to the 10X Genomics Chromium Single Cell 3' Reagent Kits User Guide. Sequencing was performed on an Illumina NovaSeq S4 at GENEWIZ (Azenta US, Inc.). The FASTQ data files were aligned to the human reference sequence (refdata-gex-GRCh38-2020-A) using 10X Genomics CellRanger on the NIH HPC Biowulf cluster. (http://hpc.nih.gov). Five liver samples of healthy donors (GSM4041150, GSM4041153, GSM4041155, GSM4041158 and GSM4041160) and 2 liver samples of AC patients (GSM4041164, GSM4041166) were obtained from the Gene Expression Omnibus (GEO) under accession GSE136103 (9).

Seurat package (version 4.0) was used to further analyze all the single cell RNA seq data (10). Cells expressing high mitochondria genes (>10%) and cells with low quality (unique feature counts over 7,500 or less than 200) were filtered out. 51,361 features across 87,105 filtered cells were normalized and highly variable genes were identified via the FindVariableFeatures function. The data were integrated with the IntegrateData function utilizing RPCA method of the Seurat package. The first 30 principal components (PC) and the resolution 0.1 were used for the clustering via FindNeighbors and FindClusters functions. Neutrophils (24,925 cells, Cluster 0) from all groups were further analyzed. Neutrophils in liver and WBCs from AC patients were merged; and neutrophils from sAH patients were merged. The neutrophils from healthy control, AC and sAH were normalized and integrated with the IntegrateData function utilizing RPCA method. The first 20 principal components (PC) and the resolution 0.1 were used for the clustering via FindNeighbors and FindClusters functions. Differential gene expression was assessed using the findMarker function. The UMAP plots, dot plot, feature plots and heatmaps were generated by R.

Bulk RNA sequencing.

Liver tissues from 7 Healthy donors, 5 AC explanted livers and 13 sAH explanted livers were collected and subjected to bulk RNA sequencing at Johns Hopkins University.

Sequence reads were analyzed on the NIH HPC Biowulf cluster (http://hpc.nih.gov) using the updated applications of Trimmomatic, Hisat2 and FeatureCounts.

Statistical analyses.

The results are expressed as the mean ± SEM. All statistical analyses were performed using GraphPad Prism software. Student t test was performed to compare data from two groups.

Study approval.

The use of liver and serum samples from patients with sAH, AC, and donor controls was approved by the Institutional Review Board (IRB00107893 and IRB00154881) at Johns Hopkins University.

Data availability.

scRNA-seq data has been deposited into the Gene Expression Omnibus (GEO) under accession GSE 255772. Bulk RNA-seq data has been deposited into the GEO database with accession number GSE143318 (5, 11).

Values for all data points can be found in the supporting data values file.

References:

1. Aguilar-Bravo B, et al. Hepatocyte dedifferentiation profiling in alcohol-related liver disease identifies CXCR4 as a driver of cell reprogramming. *Journal of Hepatology.* 2023;79(3):728-40.
2. Cambier S, et al. The chemokines CXCL8 and CXCL12: molecular and functional properties, role in disease and efforts towards pharmacological intervention. *Cell Mol Immunol.* 2023;20(3):217-51.
3. Wieser V, et al. Reversal of murine alcoholic steatohepatitis by pepducin-based functional blockade of interleukin-8 receptors. *Gut.* 2017;66(5):930-8.
4. Zheng M, et al. Therapeutic Role of Interleukin 22 in Experimental Intra-abdominal Klebsiella pneumoniae Infection in Mice. *Infect Immun.* 2016;84(3):782-9.
5. Ma J, et al. Distinct histopathological phenotypes of severe alcoholic hepatitis suggest different mechanisms driving liver injury and failure. *J Clin Invest.* 2022;132(14):e157780.
6. Crabb DW, et al. Standard Definitions and Common Data Elements for Clinical Trials in Patients With Alcoholic Hepatitis: Recommendation From the NIAAA Alcoholic Hepatitis Consortia. *Gastroenterology.* 2016;150(4):785-90.
7. Crabb DW, et al. Diagnosis and Treatment of Alcohol-Associated Liver Diseases: 2019 Practice Guidance From the American Association for the Study of Liver Diseases. *Hepatology.* 2020;71(1):306-33.
8. Schindelin J, et al. Fiji: an open-source platform for biological-image analysis. *Nat Methods.* 2012;9(7):676-82.
9. Ramachandran P, et al. Resolving the fibrotic niche of human liver cirrhosis at single-cell level. *Nature.* 2019;575(7783):512-8.
10. Hao Y, et al. Integrated analysis of multimodal single-cell data. *Cell.* 2021;184(13):3573-87 e29.
11. Hyun J, et al. Epithelial splicing regulatory protein 2-mediated alternative splicing reprograms hepatocytes in severe alcoholic hepatitis. *J Clin Invest.* 2020;130(4):2129-45.

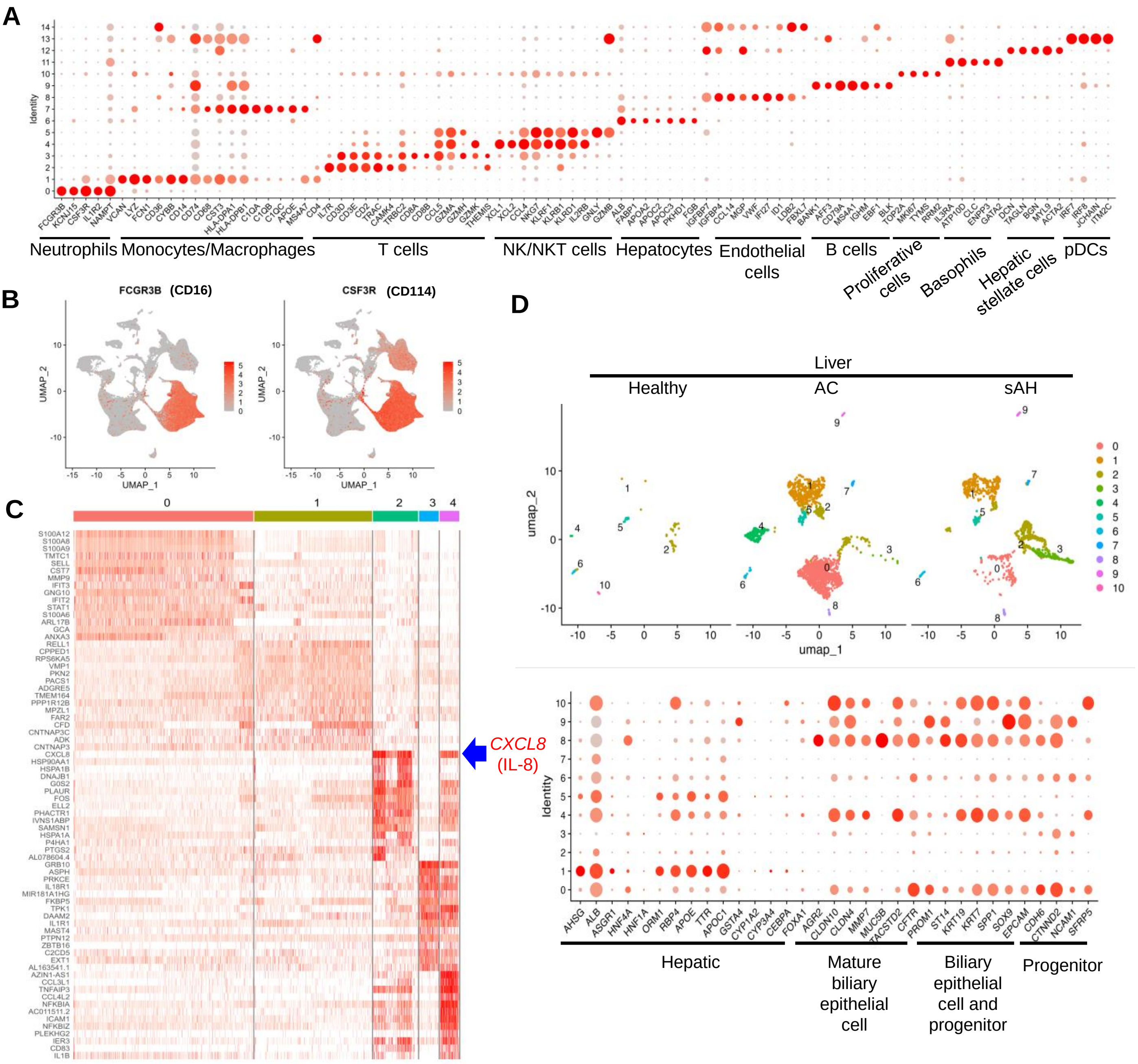

Supplemental Figure 1. ScRNA sequencing data of liver cells from Healthy donors, AC and sAH; and white blood cells from AC and sAH were analyzed, integrated and clustered by Seurat.

(A) Dot plots of the signature genes which define the specific cell types of each cluster among all groups of the scRNA data.
(B) Feature plots for the gene expression of *CD16* and *CD114*. CD16 also known as Fc Gamma Receptor IIIb (FCGR3B), CD114 also known as the granulocyte colony-stimulating factor receptor (G-CSF-R) or CSFR3.
(C) Heatmaps of the signature genes of each neutrophil cluster.
(D) A limited number of hepatic cells (hepatocyte cluster 6 in Fig. 1A) that express hepatic, biliary, and progenitor cell markers were re-clusterd and analyzed.

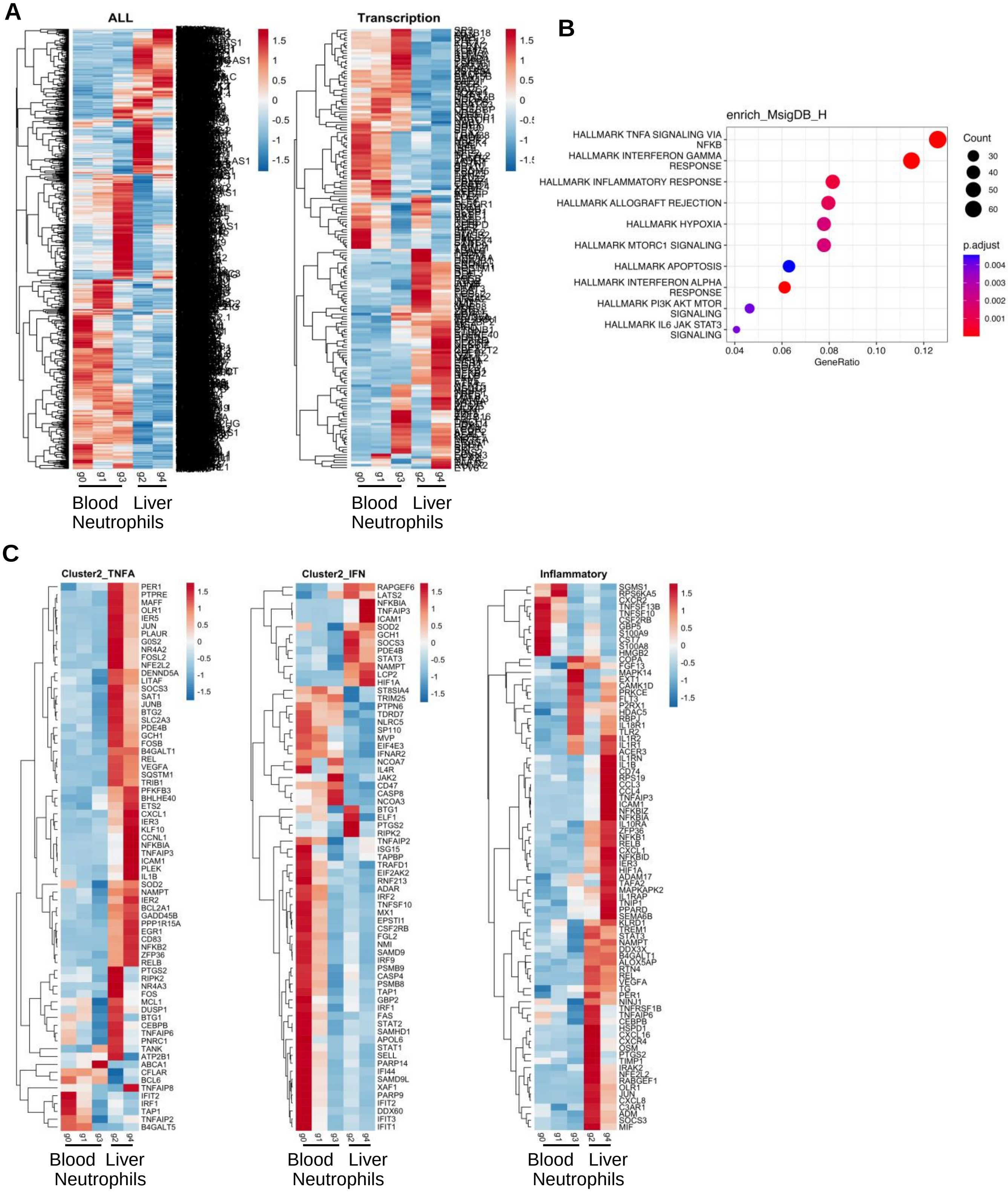

Supplemental Figure 2

(A) Heatmap of all differentially expressed genes, and transcription factor-related genes among different neutrophil clusters from Fig. 1B.

(B) Hallmark pathway analysis of differentially expressed genes among neutrophil clusters from Fig. 1B.

(C) Heatmap of TNF-α, IFN-γ, and inflammatory response related genes from the pathway analysis.

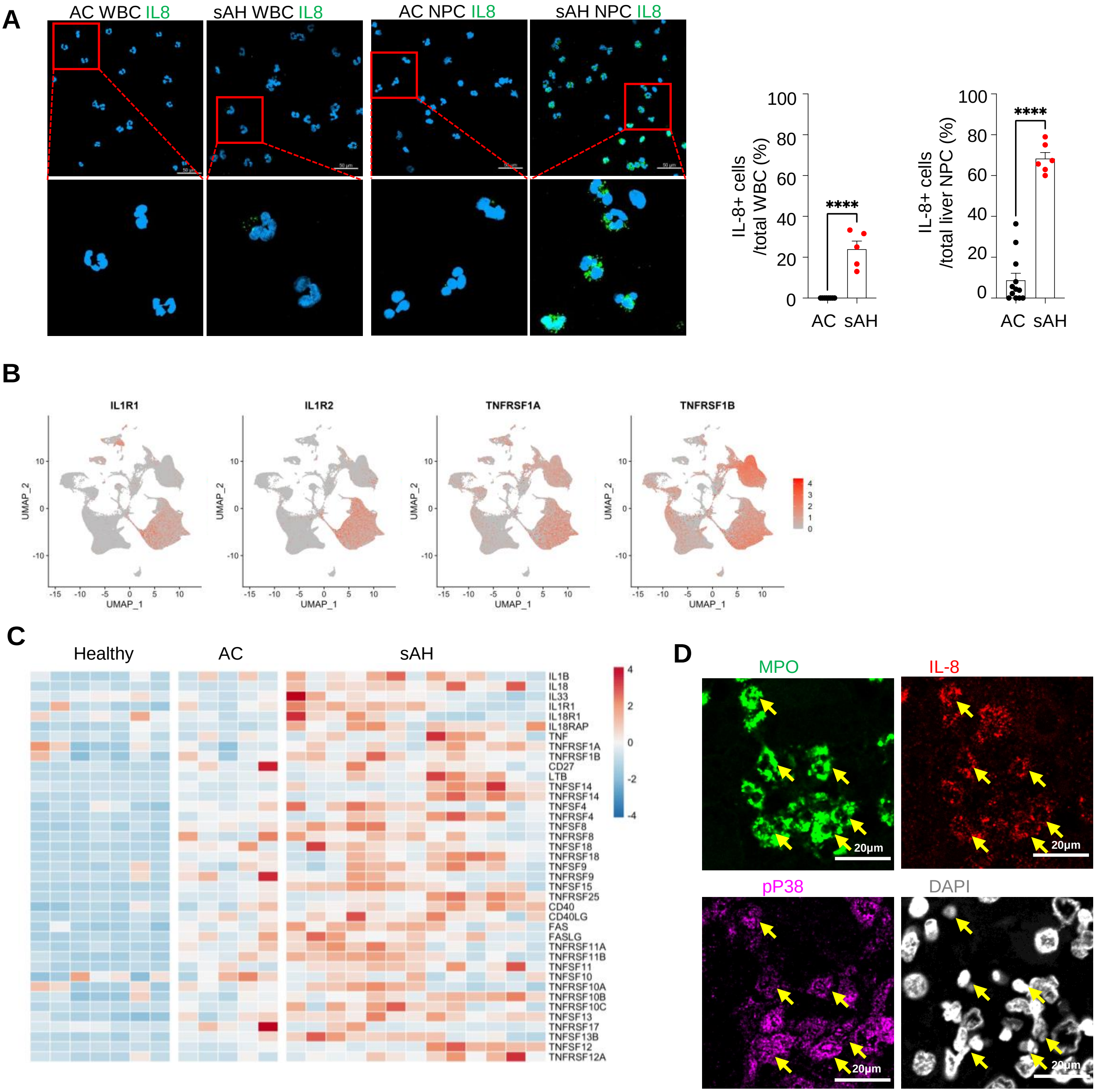

Supplemental Figure 3

(A) NPC and WBC samples form sAH and AC patients were fixed on slides by Cytospin and stained with IL-8. Representative images are shown. The number of IL-8⁺ cells was quantitated. The percentage of IL-8⁺ cells were presented as means ± SEM. ****$P < 0.0001$

(B) Feature plots of all groups of scRNA-seq data showing the gene expression of *IL1R1, IL1R2, TNFR1 (TNFRSF1A)* and *TNFR2 (TNFRSF1B)* genes among all the cells.

(C) Liver tissues from Healthy controls (Healthy), AC and sAH patients were collected and subjected to bulk RNA sequencing. Heatmap of bulk RNA-seq data for the gene expression of *IL1B* and *TNF* gene families.

(D) Representative multiplex immunofluorescence staining of pP38, MPO, and IL-8 in sAH liver tissues. The merged image is shown in Fig. 1F.

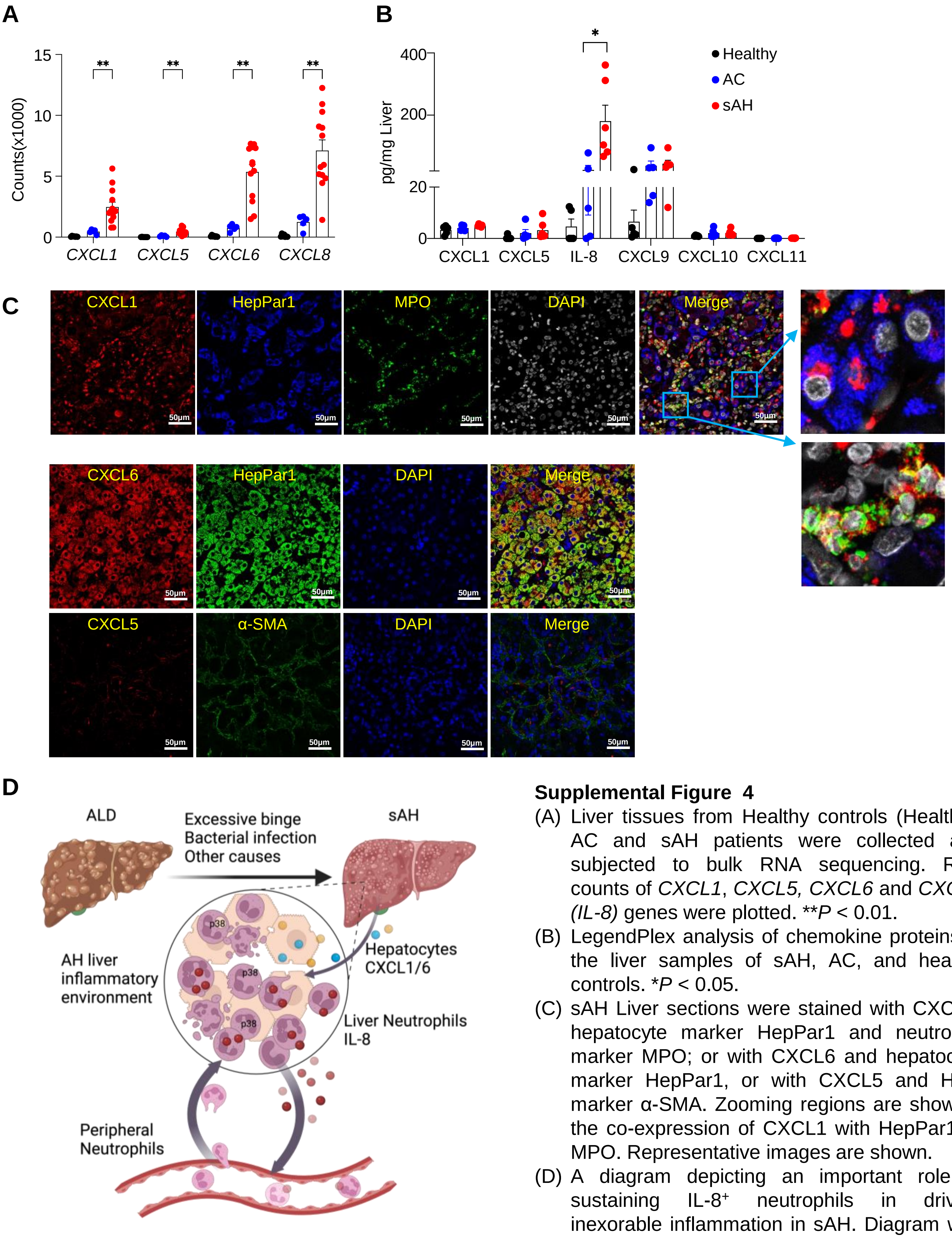

Supplemental Figure 4

(A) Liver tissues from Healthy controls (Healthy), AC and sAH patients were collected and subjected to bulk RNA sequencing. Raw counts of *CXCL1*, *CXCL5, CXCL6* and *CXCL8 (IL-8)* genes were plotted. ***P* < 0.01.

(B) LegendPlex analysis of chemokine proteins in the liver samples of sAH, AC, and healthy controls. **P* < 0.05.

(C) sAH Liver sections were stained with CXCL1, hepatocyte marker HepPar1 and neutrophil marker MPO; or with CXCL6 and hepatocyte marker HepPar1, or with CXCL5 and HSC marker α-SMA. Zooming regions are showing the co-expression of CXCL1 with HepPar1 or MPO. Representative images are shown.

(D) A diagram depicting an important role of sustaining IL-8$^+$ neutrophils in driving inexorable inflammation in sAH. Diagram was created with BioRender.com
